# In this month’s Bulletin

**DOI:** 10.2471/BLT.24.000924

**Published:** 2024-09-01

In the editorial section, Megan B. Diamond et al. (622) outline policy approaches for global wastewater surveillance. Leshan Xiu and Kun Yin (623) discuss the use of wastewater surveillance to monitor and control H5N1 influenza.

Gary Humphreys (626–627) writes on assisted dying and the role of palliative care clinicians. Alka Dwivedi talks to Gary Humphreys (628–629) about the development of a new form of CAR T-cell therapy to improve cancer treatment outcomes and lower costs.

## India

### Universal health coverage

Arnab Mukherji et al. (630–638) present district-level monitoring efforts.

## Mali

### Community health worker home visits versus fixed-site care 

Jenny Liu et al. (639–649) do a cluster-randomized trial.

## Somalia

### Vaccine delivery in fragile and conflict-affected settings 

Muhammad Farid et al. (674–680) describe a vaccination campaign for COVID-19.

## United Republic of Tanzania

### Repairs and maintenance of medical devices

Ally Kebby Abdallah et al. (665–673) detail steps taken to improve management.

## Global

### Health worker protests 

Kartik Sharma et al. (650–656) analyse event data from 2018-2022.

### Better treatment for drug-resistant tuberculosis 

Tom A Yates et al. (657–664) make the case for a phase III platform trial.

